# Supplementary material for: In situ and remote observations of the ultraviolet footprint of the moon Callisto by the Juno spacecraft
Source: Nat Commun. 2025 Sep 1;16:7791. doi: 10.1038/s41467-025-62520-4 (PMC12402282; doi:10.1038/s41467-025-62520-4)
Supplement: Supplementary file 1 — Supplementary Information [file 41467_2025_62520_MOESM1_ESM.pdf]

Supplementary Information for

“In situ and remote observations of the ultraviolet footprint of the moon Callisto by the Juno spacecraft”

J. Rabia[1,*], V. Hue[2], C.K. Louis[3], N. André[1,4], Q. Nénon[5], J.R. Szalay[6], R. Prangé[3], L. Lamy[3,7], P. Zarka[3], B. Collet[7], F. Allegrini[8,9], R.W. Ebert[8,9], T.K. Greathouse[8], B. Bonfond[10], G.R. Gladstone[8], A.H. Sulaiman[11], W.S. Kurth[12], J.E.P. Connerney[13,14], P. Louarn[1], E. Penou[1], A. Kamran[1], D. Santos-Costa[8], R.S. Giles[8], J.A. Kammer[8], M.H. Versteeg[8], and S.J. Bolton[8]

[1]Institut de Recherche en Astrophysique et Planétologie (IRAP), CNRS, CNES, Toulouse, France
[2]Aix-Marseille Université, Institut Origines, LAM, Marseille, France
[3]LIRA, Observatoire de Paris, Université PSL, Sorbonne Université, Université Paris Cité, CY Cergy Paris Université, CNRS, 92190 Meudon, France
[4]ISAE-Supaero, Université de Toulouse, Toulouse, France
[5]Laboratoire Atmosphère Observations Spatiales, CNRS- Sorbonne Université–CNES, Paris, France
[6]Department of Astrophysical Sciences, Princeton University, Princeton, NJ, USA
[7]Aix-Marseille Université, CNRS, CNES, LAM, Marseille, France
[8]Southwest Research Institute, San Antonio, TX, USA
[9]Department of Physics and Astronomy, University of Texas at San Antonio, San Antonio, TX, USA
[10]Laboratory for Planetary and Atmospheric Physics, University of Liège, Liège, Belgium
[11]School of Physics and Astronomy, Minnesota Institute for Astrophysics, University of Minnesota, Minneapolis, MN, USA
[12]Department of Physics and Astronomy, University of Iowa, Iowa City, IA, USA
[13]Space Research Corporation, Annapolis, MD, USA,
[14]NASA/Goddard Space Flight Center, Greenbelt, MD, USA
*Contact: jonas.rabia@irap.omp.eu

This document includes:

- Supplementary Figure 1 to Supplementary Figure 8
- Supplementary Table 1
- Supplementary References

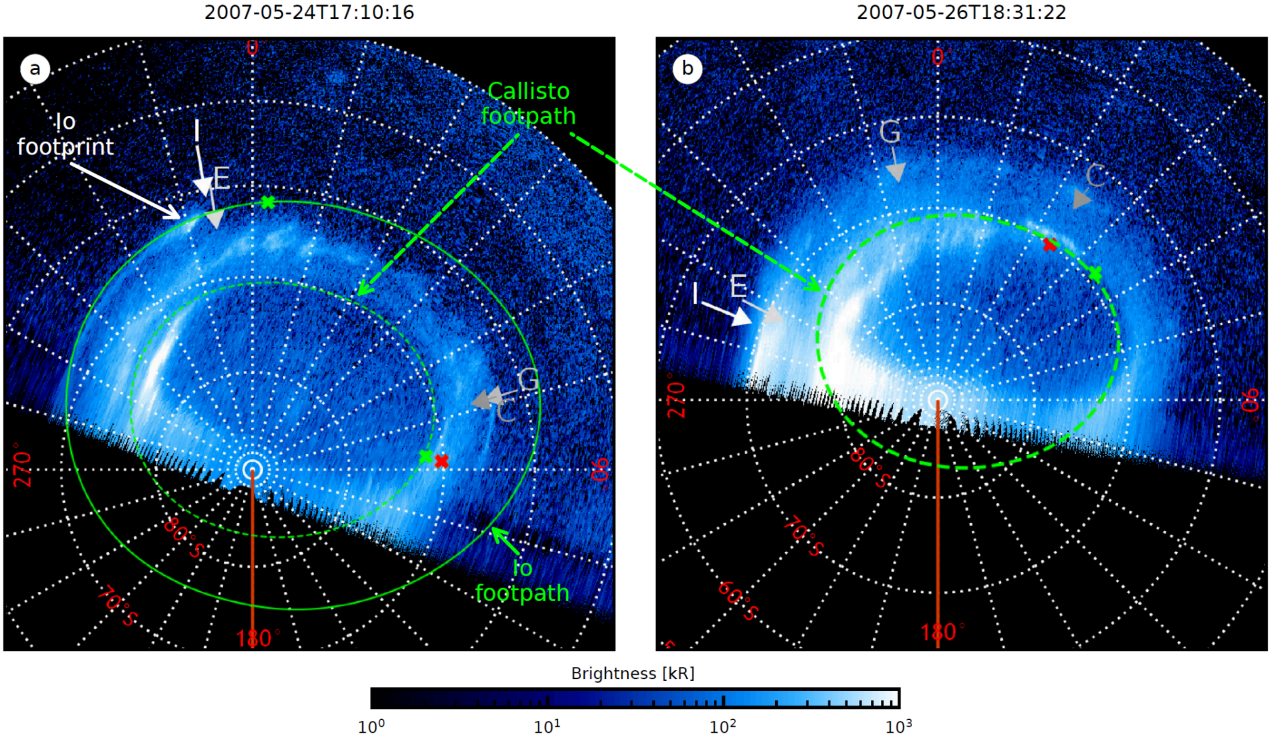

Supplementary Figure 1 | Previous reported detections of the Callisto footprint. *Hubble Space Telescope observations of Jupiter's southern hemisphere on 2007-05-24 (a) and 2007-05-26 (b). Green dashed lines indicate the Callisto footpath derived using JRM33+KK2005, while the plain green line in panel (a) shows the Io footpath, computed with the JRM33+CON2020 model. Instantaneous moon magnetic footprints calculated by the ISaAC[1] magnetic field model at the time indicated on the panel titles are highlighted by white-gray arrows while those calculated by the JRM33+CON2020 (Io) and JRM33+KK2005 (Callisto) are indicated by green crosses. Locations of the auroral spots identified as Callisto-induced aurora by ref.2 are indicated by red crosses.*

In panel (a), a latitude separation (~1.7°) is observed between the estimated location and the identified footprint. This can be explained by uncertainties in the magnetic field mapping and/or uncertainties on the fitting of the footprint position or the detection of a UV auroral structure not related to Callisto.

A slightly smaller latitude separation is also observed in panel (b). However, in that case, the longitude separation between Callisto's instantaneous footprint and the spot identified as Callisto-induced auroral emission, i.e., the lead angle, is estimated to be ~15°. This large lead angle, that is proportional to the plasma density in the moon's vicinity[3], is poorly consistent with the low plasma density existing at the Callisto orbital location. In this case, the longitude discrepancy can be explained by uncertainties of the magnetic field model, or the detection of a UV auroral structure not related to Callisto.

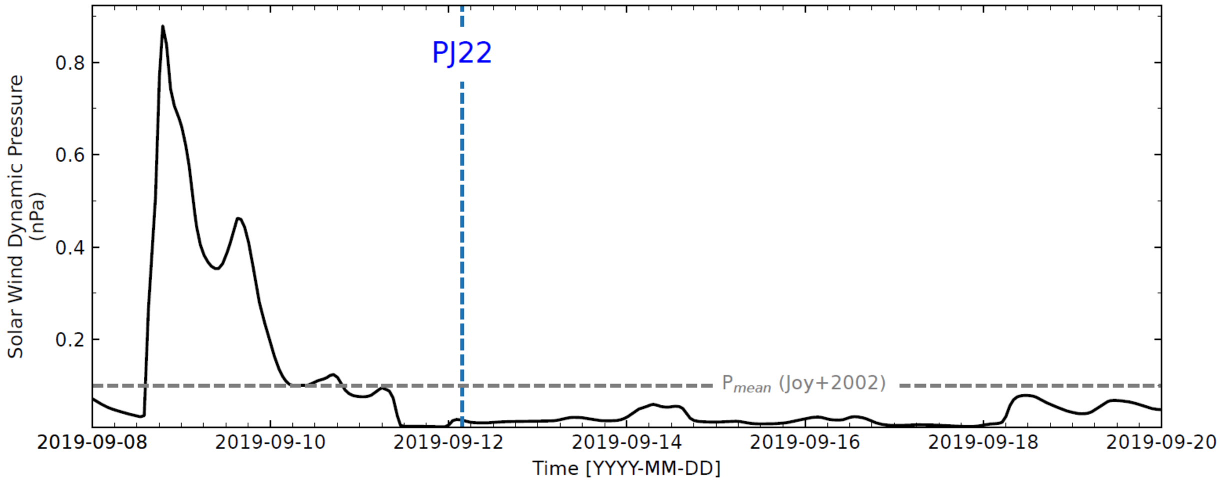

Supplementary Figure 2 | Solar Wind Dynamic Pressure at Jupiter around Juno Perijove 22. *Evolution of the Solar Wind Dynamic Pressure (black curve) around Perijove 22 and Callisto's flux tube crossing (blue dashed line). The mean value of the solar dynamic pressure at Jupiter derived by ref.4 is indicated by a horizontal gray dashed line. Tao's model[5] with OMNI data as input was used for the propagation of the Solar Wind. The angle between Jupiter, the Sun and the Earth is in the range [64°-76°] during this interval, therefore, the confidence level of the Tao model is high.*

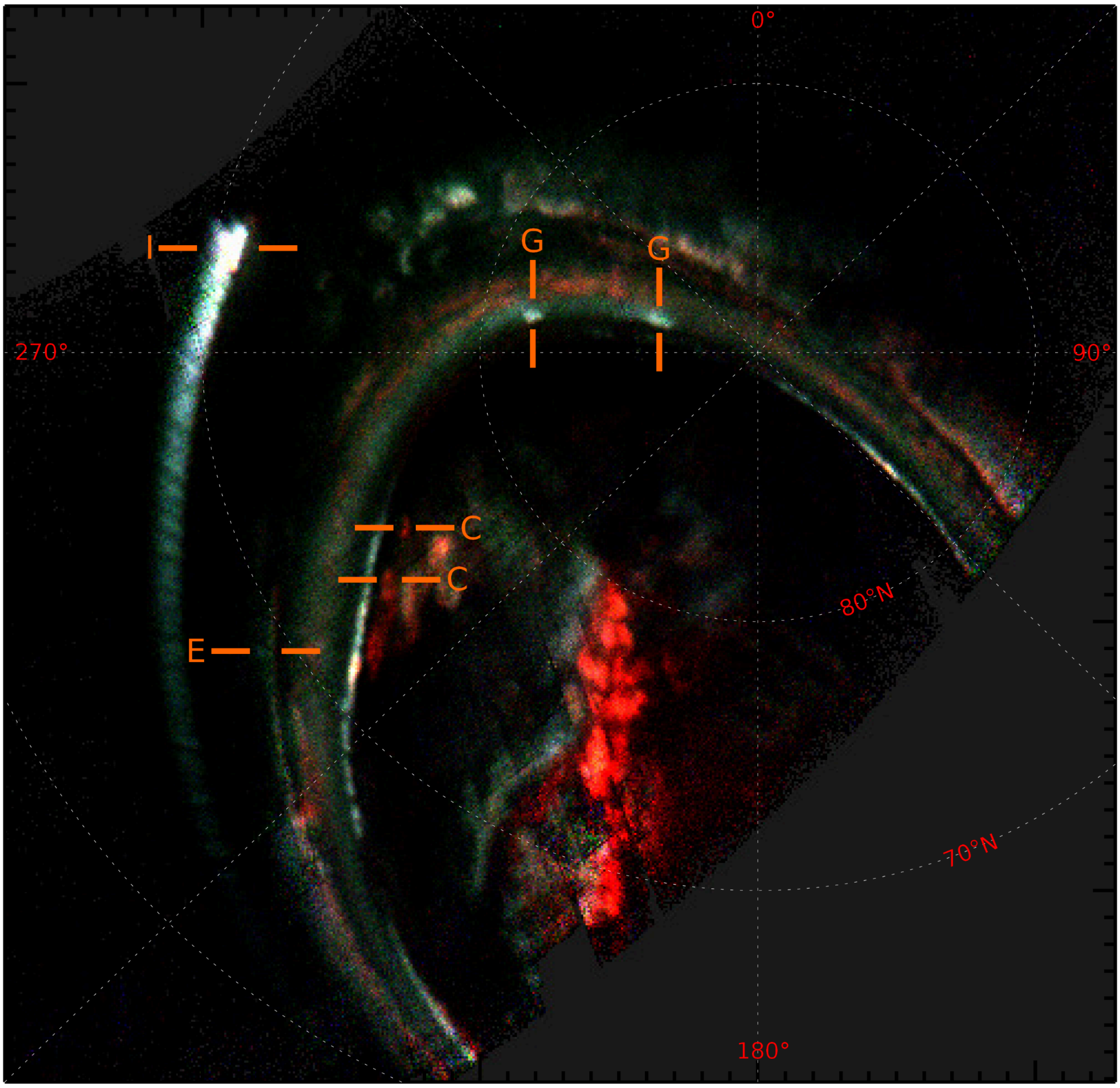

Supplementary Figure 3 | Juno-UVS observations of the northern auroral region of Jupiter during PJ22. *False color UV map of the auroral structures observed onto Jupiter's northern auroral region resulting from co-adding consecutive Juno-UVS data from 02:54:00 to 03:09:02. The colors represent various UV spectral bands: red, green, and blue tend to correspond to high-, medium-, and low-energy electron precipitation, respectively, while white indicates a mixture of energies. The UV footprints of Io (I), Europa (E), Ganymede (G), and Callisto (C) are highlighted between orange lines.*

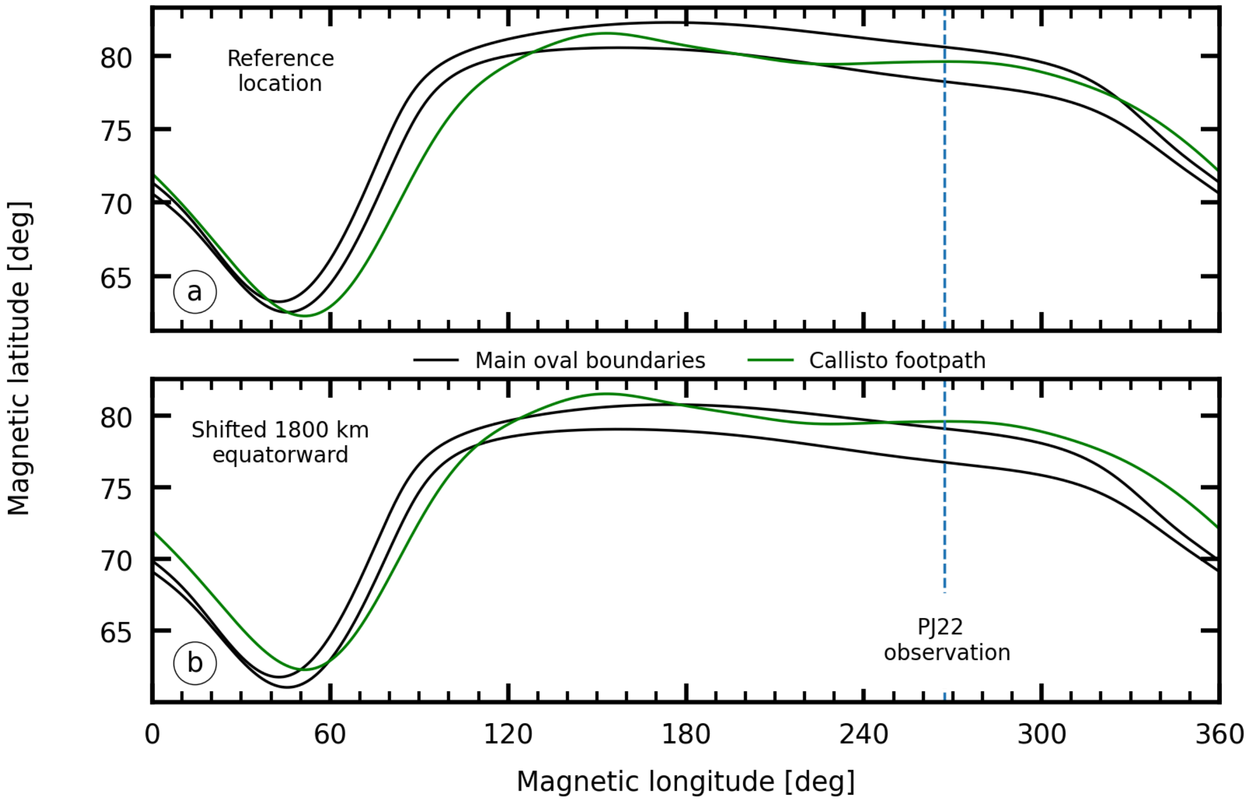

Supplementary Figure 4 | Location of Callisto's auroral footprint with respect to the main oval emissions. a, *The location of the Callisto footpath in the northern auroral region is compared to the reference outer and inner limits of the main auroral emission in the northern hemisphere. In most of the longitude sectors, the Callisto footpath is co-located with the main auroral emissions.* ***b****, The main oval boundaries are shifted by 1800 km, ~ 1.5°, equatorward, corresponding to the shift observed during PJ22N[6]. As the Io, Europa, and Ganymede footprint do not exhibit a significant shift from their expected locations, we consider that the Callisto footpath is not shifted as well. Magnetic longitude and latitude are calculated in the JRM33 reference frame, i.e., a coordinate system tilted by 10.25° towards* λ_{III} *= 196.38° with respect to the* S_{III} *reference frame.*

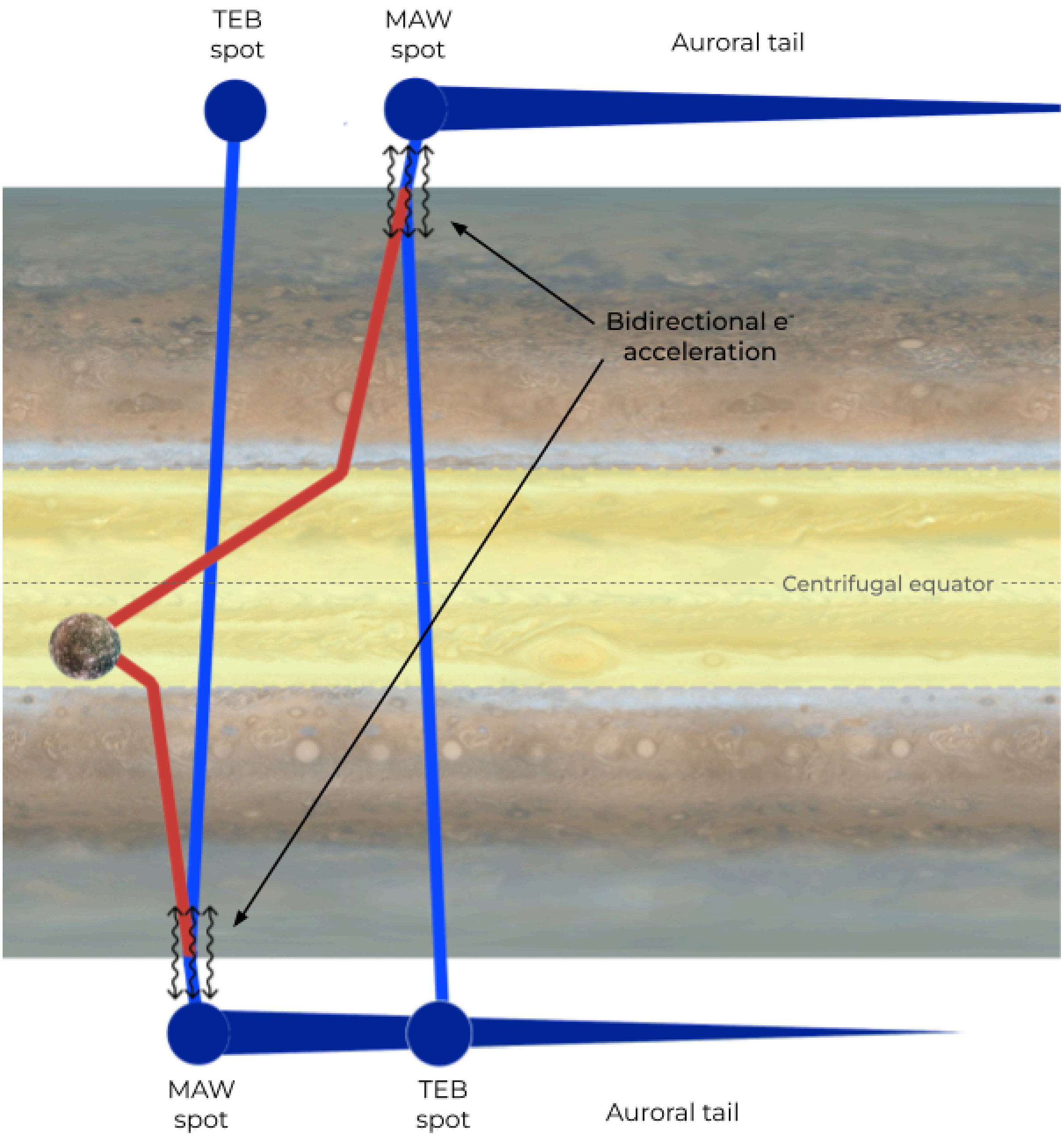

Supplementary Figure 5 | Illustration of the TEB and MAW spot generation process. *Adapted from Bonfond et al. (2008, doi: 10.1029/2007GL032418). During PJ22 observations, Callisto is located below the centrifugal equator. As a result, the Alfvén waves propagate faster towards Jupiter's south pole than towards the north (red lines), as they encounter a region of high plasma density and low Alfvén speed, i.e., the magnetodisc (yellow region). Close to Jupiter, bidirectional electron acceleration occurs, leading to the formation of the MAW spot in one hemisphere and of the TEB spot in the opposite one. Credit Jupiter's surface image: NASA/JPL/Space Science Institute*.

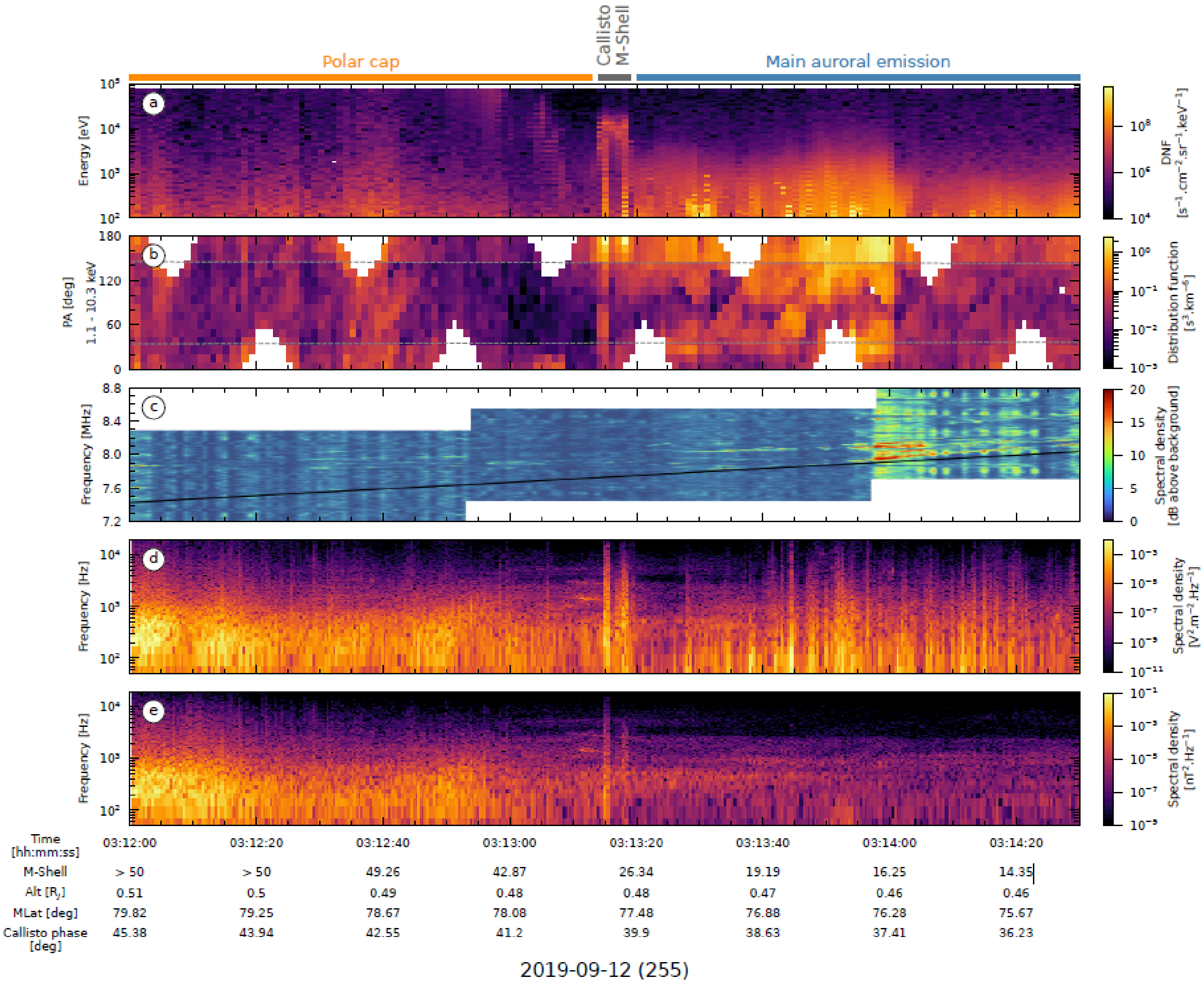

Supplementary Figure 6 | JADE-E and Waves observations during the Callisto flux tube crossing. a, *Electron energy-time spectrogram. Same as Fig 3a.* ***b,*** *Pitch angle-time spectrogram for electrons within energy ranges of 1.1 - 10.3 keV. Flux intensity is expressed in distribution function unit ($s^3.km^{-6}$).* ***c,*** *Juno-Waves observations in the HFR-Hi channel. The electron cyclotron frequency f_{ce} is indicated by the black plain line. No clear observation of decametric radio emissions is visible close to f_{ce} during the flux tube crossing.* ***d,e,*** *Frequency-time spectrogram of electric and magnetic spectral densities measured by the LFR-Lo and LFR-B channels.*

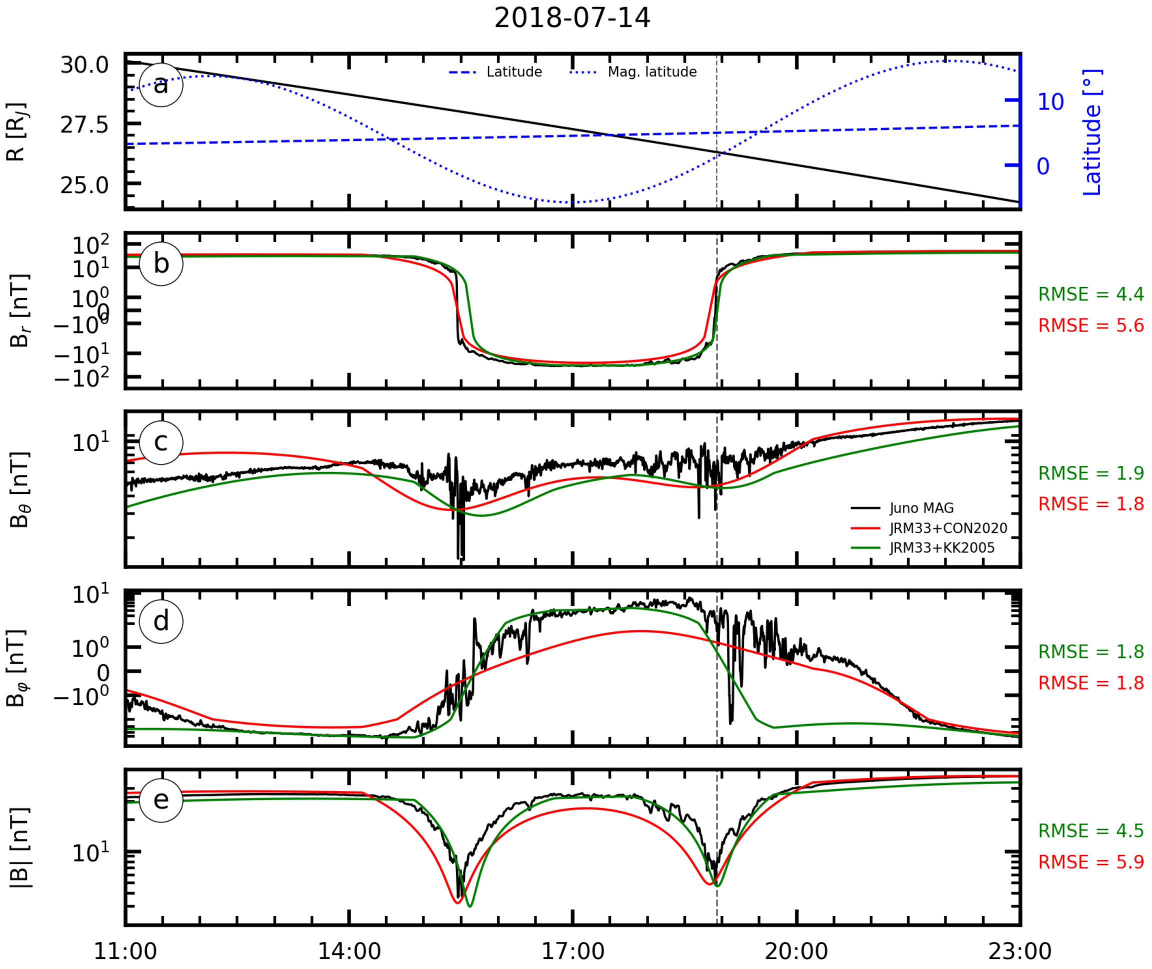

***Supplementary Figure 7 | Estimates of the magnetic field near the orbit of Callisto**. Juno-MAG measurement obtained close to the Callisto orbit during PJ14 compared to the magnetic field model estimates. **a,** Juno radial distance, magnetic, and jovigraphic latitude. **b,c,d,e** B_r, B_θ,B_Φ, |B| magnetic field components from the Juno-MAG instrument (black) compared to estimates by JRM33+CON2020 (red) and JRM33+KK2005 (green). Root mean square error (RMSE) quantifying the accuracy of the magnetic field model estimates is indicated for each magnetic field component.*

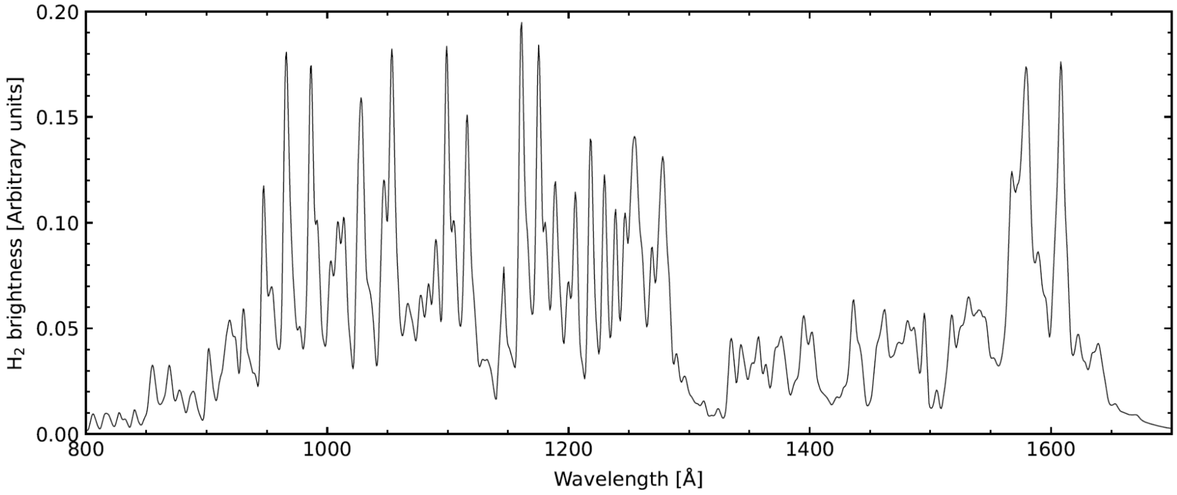

Supplementary Figure 8 | H_2 synthetic spectrum used to calculate the UV brightness. *This synthetic spectrum was simulated by accounting for the Lyman, Werner and Rydberg band systems of H2, assuming 300K for the rotational and vibration H2 temperatures, and excited by a mono-energetic electron beam of 100 eV. The spectrum was simulated as non-absorbed by the Jovian stratospheric hydrocarbons, and self-absorption was not accounted for.*

	Juno	Io	Europa	Ganymede	Callisto
Longitude λ_{III} [deg]	265.9	318.1	295.4	14.6	306.8
Magnetic lat. θ_{mag} [deg]	78.0	-5.4	-1.4	-10.2	-3.8
Centrifugal lat. θ_{cent} [deg]	77.1	-3.0	-0.4	-8.3	-2.8
Local time [h]	15.22	11.7	13.3	8.0	12.5

Supplementary Table 1 | ***Relevant orbital parameters at 2019-09-12T03:13:00.***

Supplementary References

1. Hess, S. L. G., Bonfond, B., Bagenal, F. & Lamy, L. A model of the Jovian internal field derived from in-situ and auroral constraints. *Planetary Radio Emissions VIII* 157–167 (2017) doi:10.1553/PRE8s157.
2. Bhattacharyya, D. *et al.* Evidence for Auroral Emissions From Callisto's Footprint in HST UV Images. *Journal of Geophysical Research: Space Physics* **123**, 364–373 (2018).
3. Hue, V. *et al.* The Io, Europa, and Ganymede Auroral Footprints at Jupiter in the Ultraviolet: Positions and Equatorial Lead Angles. *Journal of Geophysical Research: Space Physics* **128**, e2023JA031363 (2023).
4. Joy, S. P. *et al.* Probabilistic models of the Jovian magnetopause and bow shock locations. *Journal of Geophysical Research: Space Physics* **107**, SMP 17-1-SMP 17-17 (2002).
5. Tao, C., Kataoka, R., Fukunishi, H., Takahashi, Y. & Yokoyama, T. Magnetic field variations in the Jovian magnetotail induced by solar wind dynamic pressure enhancements. *Journal of Geophysical Research: Space Physics* **110**, (2005).
6. Head, L. A. *et al.* Effect of magnetospheric conditions on the morphology of Jupiter's ultraviolet main auroral emission as observed by Juno-UVS. *A&A* (2024) doi:10.1051/0004-6361/202450253.
